# Increasing proactive co-dispensing of take-home naloxone with prescription opioids at Ontario community pharmacies

**DOI:** 10.1177/17151635221102415

**Published:** 2022-06-09

**Authors:** Autumn Qiu Hua Chen

**Affiliations:** Autumn Chen is a PharmD student at the University of Toronto

Opioid-induced respiratory depression is a public health crisis.^1^ In 2019 alone, the Public Health Agency of Canada reported 3,669 apparent opioid toxicity deaths and 4,514 hospitalizations due to opioid-related poisoning in Canada.^1^ In Ontario, about one-third to one-fourth of opioid-related deaths involved prescription opioids.^2^ This presents an area for possible intervention by means of take-home naloxone (THN) distributed from pharmacies that are dispensing opioids. In Canada, THN is available in most pharmacies and is publicly funded in provinces such as Ontario. Canada’s Drug and Health Technology Agency (CADTH) conducted a systematic review in 2019, and key findings demonstrated that THN was associated with a reduction in overdose mortality and was cost-effective in the population of people who use heroin.^3^ Despite this, an Ontario study found that only 40.7% of patients on prescription opioid agonist therapy and 1.6% of patients on prescription opioids received THN.^2^ As a pharmacy student working in community pharmacy, I saw that naloxone dispensing was inadequate. I was utterly confused the first time a patient asked me for THN. I was unaware of how to process this request through the pharmacy management system and where the THN was located. Naloxone was so rarely dispensed that it had not been included in the regular onboarding training.

Given the stigma surrounding opioid and naloxone use, as well as the difficulties in assessing the risk of opioid-induced respiratory depression, current guidelines suggest that “all patients receiving an opioid should be dispensed take-home naloxone and counselled by a pharmacist.”^4^ Pharmacists can play an essential role in combating this public health crisis: by co-dispensing naloxone with opioids, we can improve patient and population health while also lowering costs associated with opioid-related management and hospitalizations. This would help to achieve the Quadruple Aim in Healthcare: better outcomes, lower costs, improved patient experiences and improved clinician experiences.^5^ This also aligns with the Ontario College of Pharmacists’ updated Quality Indicators for Pharmacy, which aims to focus on opioid management, reduce hospital visits for opioid poisonings among patients who are actively treated with an opioid prescription and improve patient and caregiver experiences and outcomes.^6^ Many patients do not perceive their need for THN or consider the risk of opioid exposure to those around them, therefore creating a need for a pharmacist-initiated intervention.

Considering this, I propose a quality improvement initiative to increase proactive co-dispensing of THN with opioids at community pharmacies. Individual pharmacies should assemble teams with varied expertise to support the project. There needs to be buy-in from the executive authority, the pharmacy manager and the pharmacy staff. Senior management support can provide resources, overcome barriers and implement necessary workflow changes to allow increased naloxone dispensing. Pharmacists must validate the opioid prescription, co-dispense naloxone and counsel patients on the safe use of opioids and administration instructions for THN. Other pharmacy staff members should interact with the patient and the pharmacy system to identify opportunities for THN distribution. For example, these staff members can be situated at the drop-off or pick-up counter and can refer a patient for a pharmacist consultation if they notice an opioid prescription without THN.

An aim statement with SMART (Specific, Measurable, Achievable, Relevant and Time-Bound) criteria is vital for a successful project. For example, the aim can be to increase the co-dispensing of THN with prescription opioids at the pharmacy from 40% to 80% in 6 months. Ways to evaluate the success of the project need to be in place, such as establishing data collection methods to report outcomes, processes and balancing measures. Interventions should be planned, completed, analyzed and reflected on for improvements. A possible intervention is to add an actionable alert for active opioid prescriptions on the pharmacy management system. Advancing further in the dispensing process would not be allowed until the pharmacist addresses the alert to co-dispense THN. However, any intervention needs to be analyzed for its advantages and shortcomings. In this case, would a THN co-dispensing alert lead to alert fatigue and desensitization? Would it be possible to minimize nuisance alerts so that pharmacists could focus on clinically relevant alerts? Another intervention could entail creating a comprehensive patient educational pamphlet about naloxone and including it with all opioid prescriptions. These pamphlets may prompt more patients to inquire about naloxone and may increase its subsequent dispensing. Regardless of the intervention selected, it must be feasible, pharmacy-specific and regularly assessed for continuous engagement.

Pharmacists have an obligation to educate patients and their caregivers on the effectiveness and safety of medication therapy. By not offering THN with opioid prescriptions, we may be jeopardizing patient safety, as opioids are associated with several adverse events, including significant sedation, respiratory depression and death. Consequently, adequate dispensing and counselling on naloxone are needed for any individual using an opioid. With a proactive pharmacist-led approach and structured implementation process, increasing rates of THN co-dispensed with prescription opioids are highly achievable. ■
